# Barrier Function and Biophysical Effects of 0.104% and 0.247% Retinol Creams in Mature Facial Skin: A Prospective Study

**DOI:** 10.3390/ijms27167205

**Published:** 2026-08-12

**Authors:** Iwona Pordąb, Julia Cieślawska, Michał Gackowski, Michał J. Kowalczyk, Małgorzata Pawłowska, Justyna Gornowicz-Porowska, Tomasz Osmałek, Marta Marzec, Izabela Nowak, Anna Kroma-Szal, Mariola Pawlaczyk

**Affiliations:** 1 Department and Division of Practical Cosmetology and Skin Diseases Prophylaxis, Faculty of Pharmacy, Poznan University of Medical Sciences, 3 Rokietnicka Street, 60-806 Poznań, Polandjustyna.gornowicz-porowska@ump.edu.pl (J.G.-P.); mariolapawlaczyk@ump.edu.pl (M.P.); 2 Doctoral School, Poznan University of Medical Sciences, 70 Bukowska Street, 60-812 Poznań, Poland; 3 Department of Pharmaceutical Technology, Poznan University of Medical Sciences, 3 Rokietnicka Street, 60-806 Poznań, Poland; 4 Department of Dermatology and Venereology, Poznan University of Medical Sciences, 49 Przybyszewskiego Street, 60-355 Poznań, Poland; michalkowalczyk@ump.edu.pl; 5 Department of Applied Chemistry, Faculty of Chemistry, Adam Mickiewicz University, 8 Uniwersytetu Poznańskiego Street, 61-614 Poznań, Poland; pawlowskamalgorzata@op.pl (M.P.); marta.dabrowska@amu.edu.pl (M.M.); nowakiza@amu.edu.pl (I.N.)

**Keywords:** retinol, retinoic acid receptors, epidermal barrier repair, dermal remodeling, skin aging, extracellular matrix, transepidermal water loss, interleukin-1α

## Abstract

Retinol, a bioactive small molecule of the vitamin A family, contributes epidermal and dermal tissue repair through retinoic acid receptor γ (RARγ)/retinoid X receptor (RXR) receptor-mediated transcriptional regulation of keratinocyte differentiation, extracellular matrix (ECM) remodeling, and barrier restoration. However, the relationship between applied concentration, tissue-level regenerative outcomes, and tolerability remains incompletely characterized. This prospective, randomized, single-blind study compared biophysical effects and tolerance of two retinol concentrations in EU-compliant facial creams, high-performance liquid chromatography (HPLC)-verified as 0.104% and 0.247% (*w*/*w*). Thirty-eight women aged 40–61 years (Fitzpatrick phototypes II–III) participated across three independent sub-studies: 28 were randomized to either concentration for 12 weeks; 5 underwent split-face ultrasound imaging (0.247% versus retinol-free control) for 8 weeks; and 5 participated in a tape stripping sub-study quantifying stratum corneum interleukin-1 alpha (IL-1α) and interleukin-1 receptor antagonist (IL-1ra) by ELISA before and after 6 weeks of 0.247% retinol treatment. Main cohort assessments included transepidermal water loss (TEWL), hydration, melanin, erythema, pH, sebum, biomechanical parameters, and wrinkle grading. Both concentrations significantly improved barrier parameters—hydration, TEWL, brightness, pH, and sebum—with no inter-group differences. In the ultrasound sub-group, 0.247% retinol increased epidermal thickness (+12.4 μm), epidermal density (+3.84%), and dermal density (+1.81%) versus control, consistent with ECM reorganization and epidermal stratification. Biomechanical parameters showed no significant changes, consistent with retinol’s remodeling timeline. Consumer assessment indicated comparable efficacy; 0.247% demonstrated superior smoothing but higher erythema incidence. In the tape stripping sub-study, IL-1α decreased in all participants (median: 25.1 → 13.2 picograms per tape [pg/Tape]; 5/5 concordant), while IL-1ra increased in 4/5 participants (median: 352.8 → 1403.1 picograms per three sequential tapes [pg/3sT]), indicating a directionally consistent shift in the IL-1α/IL-1ra balance; these results are exploratory and require replication in larger cohorts. These findings collectively support clinically meaningful barrier restoration and structural remodeling within current EU safety limits (Commission Regulation EU 2024/996), with 0.104% offering a favorable efficacy-to-tolerability profile for initial therapy.

## 1. Introduction

Cutaneous tissue homeostasis relies on coordinated regenerative processes, including keratinocyte proliferation and differentiation, fibroblast activation, extracellular matrix (ECM) synthesis, and epidermal barrier maintenance [1,2,3]. Achieving clinically meaningful tissue-level improvements in aged or damaged skin requires sustained modulation of these processes over extended periods, as structural remodeling and functional restoration occur through cumulative molecular changes rather than acute interventions [4,5,6]. This temporal requirement presents a fundamental challenge in dermatological therapeutics: active ingredients must not only penetrate target tissue layers and reach bioactive concentrations [7,8,9], but also maintain consistent molecular signaling over weeks to months to induce measurable structural and functional outcomes [9,10]. Among topically applied agents, retinoids have demonstrated a unique capacity to fulfill these criteria. They exhibit sustained bioactivity through enzymatic conversion to transcriptionally active metabolites, progressive accumulation in target compartments, and dose-dependent modulation of gene expression programs governing epidermal renewal and dermal ECM organization [11,12,13,14,15].

Retinol, a vitamin A derivative, is a small molecule with well-documented regenerative effects on human skin [16,17]. Its mechanism of action is based on binding to retinoic acid receptors (RARs), predominantly the RARγ subtype in the epidermis [11,16]. Once retinol penetrates the cell, it undergoes metabolic conversion via enzymes such as lecithin retinol acyltransferase (Lrat), retinol dehydrogenase 12 (Rdh12), and aldehyde dehydrogenase 1 family member A3 (Aldh1a3) to retinoic acid (RA), the most biologically active metabolite, activating signaling pathways responsible for skin regeneration, barrier repair, and ECM remodeling [17,18,19]. A one-year proteomic study showed that long-term retinol use modulates 217 stratum corneum proteins, with retinol and RA sharing about 65% of these changes [20]; among them, RA alters the tight junction proteins claudin-1 and claudin-4, reflecting its role in epidermal barrier reorganization during keratinocyte differentiation [21]. In the dermis, retinol stimulates fibroblasts to produce collagen types I and III via the transforming growth factor-beta (TGF-β)/connective tissue growth factor (CTGF) pathway, along with fibronectin, tropoelastin, and hyaluronic acid [22,23,24]. Retinol also modulates melanogenic enzymes, including tyrosinase and tyrosinase-related protein 1/2 (TRP-1/TRP-2), involved in the regulation of skin pigmentation [25].

Skin aging represents a primary clinical indication for retinoid-based regenerative therapy. This multifactorial process is driven by cellular senescence, oxidative stress, immune dysregulation, and the interplay of intrinsic and extrinsic factors, inducing progressive structural and functional changes across all skin layers [26,27,28]. Histologically, aging manifests as epidermal and subcutaneous thinning, dermal-epidermal junction flattening, reduced cellular turnover, impaired barrier function [29,30], and ECM degradation driven by elevated matrix metalloproteinase (MMP) activity [31]. Ultraviolet (UV) radiation remains the dominant extrinsic factor, responsible for a substantial proportion of visible aging signs including wrinkles, hyperpigmentation, and loss of elasticity [31,32].

Retinol exhibits superior skin penetration and more rapid conversion to RA than its esterified forms, such as retinyl palmitate, which show minimal enzymatic conversion and lower dermatological potency [33]. Bioconversion of topical retinol to RA within skin is saturable and primarily governed by enzymatic activity and cellular binding capacity rather than applied dose, suggesting that moderate concentrations may achieve clinical outcomes comparable to higher doses while reducing irritation risk [18,34]. Supporting this concept, a comparative in vivo study demonstrated that 0.3% and 1% retinol were equally effective at inducing keratinocyte proliferation, fibrillin-rich microfibril deposition, and epidermal thickening in photodamaged skin, while 0.3% retinol was significantly better tolerated, with fewer and milder adverse events [35].

Based on the 2022 Scientific Committee on Consumer Safety (SCCS) opinion on vitamin A safety, Commission Regulation (EU) 2024/996 restricts maximum retinol concentrations to 0.05% in body products and 0.3% in facial leave-on and rinse-off products [36,37].

The effects of retinol use depend on therapy duration and tissue depth. Maximum retinol-induced molecular changes in the epidermis are observed within the first 3–6 months of treatment, whereas measurable structural and clinical improvements, including dermal matrix remodeling, become evident after approximately 12 months [14,15,20]. The most pronounced molecular changes occur during initial treatment stages (first three months), whereas clinical effects such as improved texture, firmness, and wrinkle reduction appear later [5,15,20]. Topical retinoid use requires caution due to risk of erythema, dryness, and burning sensations (retinoid dermatitis), as well as increased UV sensitivity, particularly during initial therapy stages, necessitating photoprotection [5,16]. Physiological lipids, especially phytosteryl/octyldodecyl lauroyl glutamate and lipid mixtures containing ceramide 3 and cholesterol, effectively mitigate retinol-induced irritation [5,16,38]. Pregnancy and lactation remain the most important contraindications due to retinoid embryopathy risk even with topical application [5,39].

Alongside structural and barrier effects, retinol may also modulate the inflammatory microenvironment of the stratum corneum. Interleukin-1 alpha IL-1α is a constitutively expressed epidermal alarmin released upon barrier disruption, while interleukin-1 receptor antagonist (IL-1ra) acts as its endogenous competitive inhibitor, limiting IL-1-driven inflammation without receptor agonism [40,41]. The IL-1ra/IL-1α ratio in the stratum corneum reflects local inflammatory tone and serves as a non-invasive readout of barrier homeostasis [41]. During early retinol treatment, transient barrier disruption may trigger IL-1α release; however, with sustained application and progressive barrier restoration, a shift toward reduced IL-1α and elevated IL-1ra would be expected as part of an adaptive anti-inflammatory response [40,42]. Minimally invasive tape stripping enables in situ quantification of these cytokines without biopsy [43,44], yet their modulation by cosmetic retinol in mature healthy skin has not been previously characterized. To address this gap, a sub-study was conducted to assess, for the first time, stratum corneum IL-1α and IL-1ra levels following 0.247% retinol treatment. This approach was designed to provide a non-invasive inflammatory readout complementary to the biophysical assessments.

Despite proven efficacy, retinoids present formulation challenges due to inherent chemical instability. Retinol and derivatives are highly susceptible to oxidation and degradation, leading to unpredictable decreases in active content over time, especially during manufacture, transport, and storage [45,46]. Current EU regulations (Commission Regulation EU 2024/996) restrict maximum retinol concentrations but do not require routine analytical verification of actual content post-manufacturing—compliance is based solely on label claims, microbiological purity, and basic physicochemical criteria [37]. Consequently, declared retinol content may not reflect true concentration available to consumers [45].

The aim of this study was to compare the efficacy, tolerance, and impact on biophysical and biomechanical skin parameters of creams intended for mature skin, containing two different retinol concentrations: 0.104% (as determined by high-performance liquid chromatography (HPLC), labeled as 0.1%) and 0.247% (as determined by HPLC, labeled as 0.3%). Additionally, a sub-study was conducted to assess, for the first time, the effect of 0.247% retinol on stratum corneum IL-1α and IL-1ra levels using minimally invasive tape stripping, providing a novel inflammatory readout complementary to the biophysical outcomes. To address the regulatory gap regarding analytical verification and ensure data accuracy, all commercial creams used in this study were independently analyzed for actual retinol content via HPLC prior to clinical testing.

## 2. Results

### 2.1. Analytical Verification of Retinol Content

HPLC analysis confirmed that the retinol concentrations in the commercial formulations differed from their labeled values. The formulation labeled as 0.3% retinol contained 0.247% ± 0.007% (*w*/*w*), while the formulation labeled as 0.1% retinol contained 0.104% ± 0.001% (*w*/*w*). Validation parameters of the HPLC method are summarized in Table A1 (Appendix A).

### 2.2. Barrier Function and Biophysical Parameters

Statistically significant improvements over time (*p* < 0.05) were observed for most investigated skin parameters following 12 weeks of treatment with both retinol concentrations (Table 1). No significant differences were detected between the 0.104% and 0.247% formulations. Full statistical details, including baseline group comparisons and two-way ANOVA results, are provided in Supplementary Tables S1–S3.

Both concentrations significantly improved all primary barrier function parameters: hydration increased, transepidermal water loss TEWL decreased, skin brightness (L*) and Individual Typology Angle (ITA°) increased, and sebum levels were reduced (all *p* ≤ 0.001). Skin pH showed a minor but statistically significant decrease in both groups (*p* = 0.002), consistent with strengthening of the epidermal acid mantle. The analysis of skin coloration parameters (a* and b*) did not reveal statistically significant changes over time (*p* = 0.768 for a*, *p* = 0.064 for b*).

### 2.3. Biomechanical Parameters

The analysis of biomechanical parameters (skin elasticity and viscoelasticity) measured by CutiScan CS 100 did not reveal statistically significant changes in any of the assessed parameters after 12 weeks of treatment with either retinol concentration (all *p* > 0.05).

### 2.4. Structural Changes Assessed by High-Frequency Ultrasound

**Figure 1:**
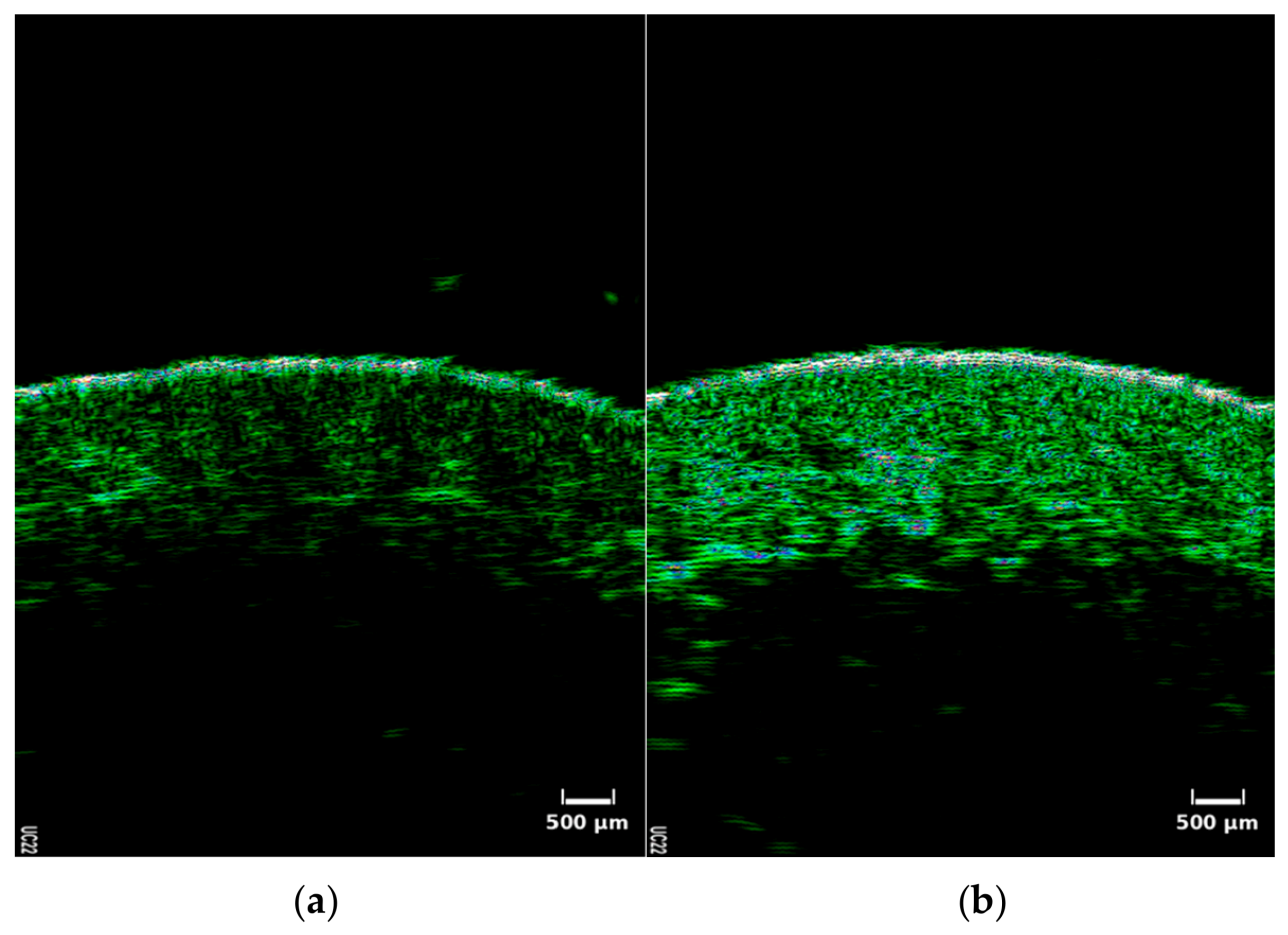
High-frequency ultrasound (22 MHz) images of facial skin at baseline (**a**) and after 8 weeks of 0.247% retinol treatment (**b**) in a representative subject, showing increased dermal echogenicity and epidermal definition. Scale bar = 500 µm.

In the split-face substudy (*n* = 5), high-frequency ultrasound (22 MHz) revealed structural changes following 8 weeks of 0.247% retinol application compared to the control side (Table 2, Figure 1). Due to the small sample size, no inferential statistics were performed; results are presented as descriptive trends.

The 0.247% retinol side demonstrated increases in epidermal thickness (+12.4 µm), epidermal density (+3.84%), and dermal density (+1.81%), while the control side remained largely stable across all parameters. Dermal thickness showed no meaningful change in either group. Representative ultrasound images (Figure 1) illustrate enhanced epidermal definition and increased dermal echogenicity following retinol treatment, consistent with epidermal stratification and ECM reorganization.

Representative ultrasound images (Figure 1) demonstrated increased dermal echogenicity and a brighter, more distinct epidermal line following retinol treatment, indicating improved collagen organization and epidermal barrier function.

### 2.5. Stratum Corneum Cytokine Profile Following Retinol Treatment

In a sub-group of five female volunteers (mean age 50.8 ± 7.0 years), tape stripping (Corneofix^®^ F20) before and after 6 weeks of 0.247% retinol treatment revealed a consistent decrease in stratum corneum IL-1α in all participants (median: 25.1 → 13.2 pg/Tape; 5/5 concordant; Wilcoxon signed-rank, exact two-sided *p* = 0.0625; Mid-*p* ≈ 0.043) and an increase in IL-1ra in 4 out of 5 participants (median: 352.8 → 1403.1 pg/3sT; *p* = 0.125; Mid-*p* ≈ 0.109). Due to the small sample size, results are presented as descriptive trends only; sampling and extraction procedures are described in Section 4.9.

### 2.6. Wrinkle Assessment

**Figure 2:**
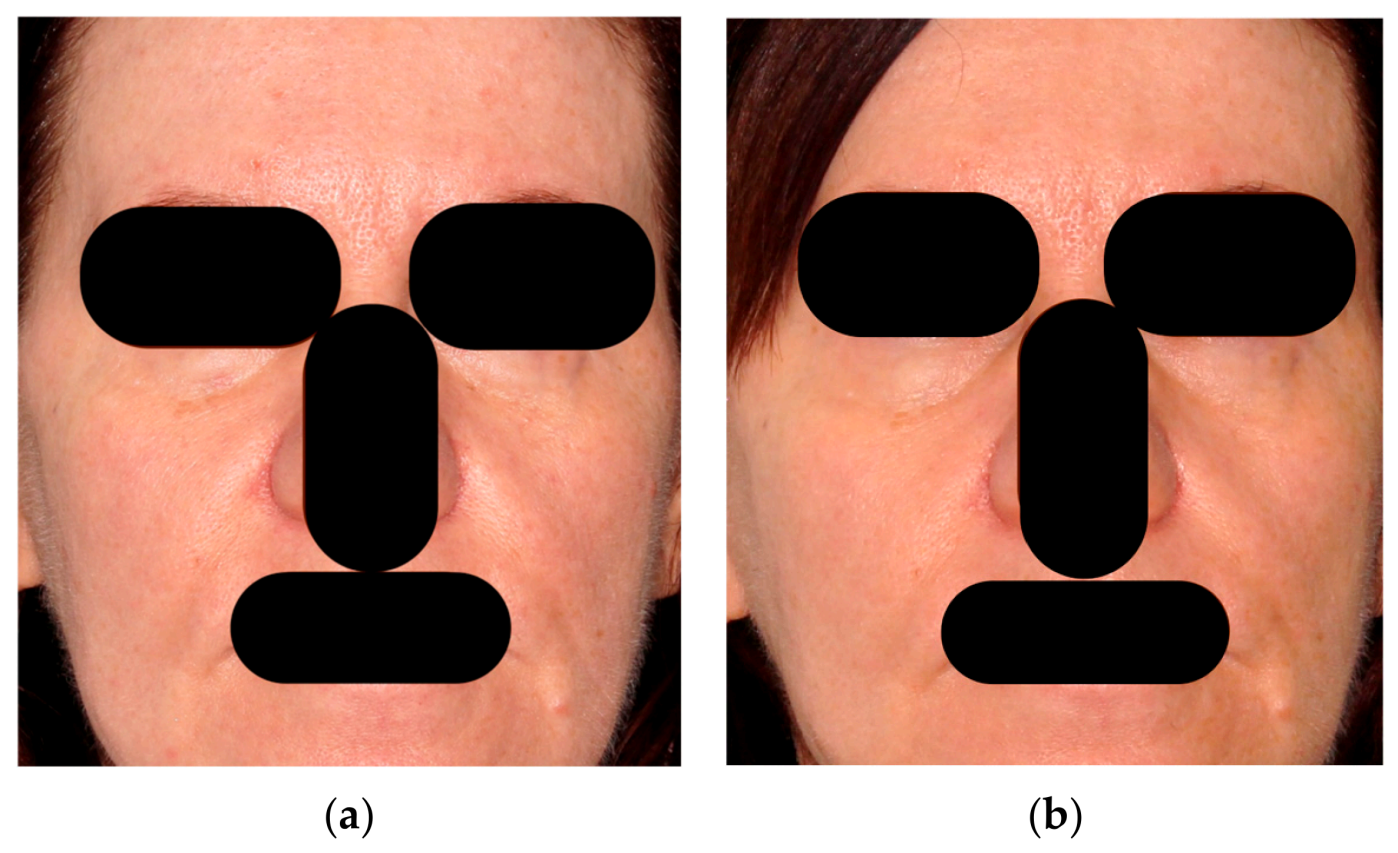
Standardized Fotofinder photographs of a female participant: (**a**) baseline image before treatment; (**b**) image after 12 weeks of topical retinol (0.247%) treatment, demonstrating visible softening of glabellar wrinkles as graded with the Skin Aging Atlas (0–5 scale), along with an improvement in overall skin tone.

Evaluation of facial wrinkles using the Skin Aging Atlas [47] protocol demonstrated small mean reductions across all assessed regions after 12 weeks of treatment in both groups combined (*n* = 28; Table 3). The greatest reductions were observed for periocular wrinkles (crow’s feet, −0.6 points) and nasolabial folds (−0.4 points), followed by glabellar wrinkles (−0.4 points), horizontal forehead wrinkles (−0.3 points), and perioral wrinkles (−0.2 points). However, none of these changes reached statistical significance (all *p* > 0.05), indicating that 12 weeks of treatment was insufficient to produce measurable wrinkle reduction by semi-quantitative grading. Representative Fotofinder photographs illustrate a visible softening trend in dynamic glabellar wrinkles after 12 weeks (Figure 2).

### 2.7. Consumer-Reported Outcomes

Consumer surveys conducted after 12 weeks revealed no significant differences between concentrations for most self-assessed parameters. However, the 0.247% retinol group reported superior skin smoothness compared to 0.104%. Transient adverse effects were more frequent in this group: overall, 6 out of 14 participants (42.9%) reported at least one adverse effect. Of these, 5 (35.7%) reported worsening of skin condition, of whom 4 (28.6%) also experienced mild irritation and erythema as part of this worsening; the remaining 1 participant (7.1%) reported dryness or reduced hydration as an isolated symptom, unrelated to the worsening/irritation cluster.

All participants reported overall satisfaction with visible improvements in skin appearance and aging signs. Nine out of 28 women reported reduced sebum production, and eight observed fewer comedones and pustules.

## 3. Discussion

### 3.1. Retinol-Mediated Tissue Remodeling: Molecular Mechanisms and Barrier Restoration

The present prospective study demonstrates that topical retinol at verified concentrations of 0.104% and 0.247% induces measurable biophysical and structural changes in mature facial skin, consistent with tissue-level remodeling and barrier repair. HPLC analysis confirmed actual retinol content of 0.104% ± 0.001% and 0.247% ± 0.007% (*w*/*w*), respectively, with the latter notably lower than the labeled 0.3%. This discrepancy reflects the inherent chemical instability of retinol, as oxidative degradation during manufacture, storage, and transport is well-documented [45,46]. These analytically verified doses enabled precise dose-outcome evaluation, addressing a critical gap in cosmetic retinoid research where declared and actual concentrations frequently diverge [45].

The observed improvements in barrier function—reduced TEWL, increased hydration, and enhanced skin brightness, and a decrease in skin surface pH—collectively reflect retinol’s capacity to modulate keratinocyte differentiation and epidermal organization [5,15,16]. In particular, the concurrent reduction in TEWL and skin surface pH points to a coordinated normalization of the acidic mantle, a key determinant of stratum corneum lipid processing and barrier competence. Retinol, following enzymatic conversion to retinoic acid (RA), activates nuclear retinoic acid receptors (RARs) and retinoid X receptors (RXRs), inducing transcriptional programs that regulate epidermal renewal, extracellular matrix (ECM) synthesis, and barrier protein expression [11,12,13]. Recent proteomic studies confirmed modulation of 217 epidermal proteins following prolonged retinol use, including those governing cornified envelope formation, lipid organization, and tight junction integrity [20].

Notably, RA alters tight junction composition by downregulating claudin-1 and upregulating claudin-4, alongside broad changes in lipid and cornified-envelope genes, reflecting a state impaired yet partially compensated barrier function during active remodeling [21]. At the biophysical level, skin surface pH significantly decreased in both groups (from 5.41 to 5.22 and from 5.45 to 5.24 in the 0.104% and 0.247% groups, respectively; *p* = 0.002). Post-treatment pH values fell within the optimal range for β-glucocerebrosidase and acid sphingomyelinase (pH~5.0–5.5), the enzymes responsible for the final steps of ceramide synthesis in the stratum corneum [48]. This supports strengthening of the acidic mantle [5,16,49].

A concomitant statistically significant reduction in sebum levels was observed in both groups after 12 weeks (*p* = 0.001). Mean baseline values (150–157 μg/cm^2^) indicated normal-to-slightly-oily, though high intra-group variability (SD 44–47 μg/cm^2^) suggests heterogeneous baseline skin types. Sebum is a key lipid component of the acid mantle, so its reduction alongside decreasing pH points to a shared mechanism: retinol-induced normalization of epidermal lipid composition [48,49,50].

### 3.2. Structural Remodeling: Ultrasound Evidence of Epidermal and Dermal Changes

High-frequency ultrasound (22 MHz) revealed increased epidermal thickness (+12.4 µm), epidermal density (+3.84%), and dermal density (+1.81%) following 8 weeks of 0.247% retinol application compared to control. These structural changes reflect tissue-level remodeling rather than superficial effects. Increased epidermal thickness indicates enhanced keratinocyte proliferation and stratification, while increased dermal density suggests ECM reorganization and collagen synthesis [14,15]. Pequeno and Bagatin confirmed that 22 MHz ultrasound provides reliable quantitative assessment of retinoid-induced structural changes, correlating echogenicity with collagen density and epidermal barrier integrity [51,52].

The molecular basis for these changes involves retinol-mediated upregulation of type I and III collagen, fibrillin, and elastin, alongside downregulation of matrix metalloproteinases (MMPs) [13,14,17]. Shao et al. demonstrated that even low retinol concentrations effectively modulate gene expression associated with ECM synthesis and keratinocyte proliferation, consistent with our findings [14]. The increased dermal echogenicity observed in our ultrasound images indicates improved collagen organization, consistent with progressive dermal regeneration [51,53].

Notably, the retinol-free control side showed only a slight decrease in epidermal density (55.19 → 54.56%) and a marginal increase in dermal density (12.00 → 12.46%), with no coordinated change across both compartments. In contrast, the 0.247% retinol side showed a concordant increase in both epidermal and dermal density, consistent with retinol’s documented mechanism of action. However, the control cream was not identical to the retinol creams minus retinol, but had an entirely different base formulation (Table 4), which limits direct comparison. Furthermore, the retinol-containing creams also contained seabuckthorn oil and ascorbyl glucoside, both listed higher than retinol in the INCI composition and therefore present at higher concentrations; as both ingredients have documented independent effects on skin structure [54,55,56], a contribution from these co-occurring ingredients to the observed structural changes cannot be excluded. 

### 3.3. Stratum Corneum Cytokine Profile: IL-1α and IL-1ra Modulation Following Retinol Treatment

The tape stripping sub-study revealed a directionally consistent decrease in stratum corneum IL-1α across all five participants following 6 weeks of 0.247% retinol treatment (median: 25.1 → 13.2 pg/Tape; 5/5 concordant; *p* = 0.0625; Mid-*p* ≈ 0.043). IL-1ra increased in 4/5 participants (median: 352.8 → 1403.1 pg/3sT; *p* = 0.125; Mid-*p* ≈ 0.109). IL-1α is constitutively expressed in keratinocytes and released upon barrier disruption as a primary epidermal alarmin, while IL-1ra acts as its endogenous competitive inhibitor [40,41,57]. In aging skin, surface IL-1ra levels decline without a corresponding change in IL-1α, shifting the IL-1ra/IL-1α ratio toward a pro-inflammatory milieu and contributing to impaired barrier recovery capacity [58,59]. The decrease in IL-1α, coinciding with improved TEWL and hydration in the main cohort, suggests progressive normalization of epidermal barrier function.

The cytokine pattern observed here—decreased IL-1α with elevated IL-1ra—is directionally consistent with profiles reported in UV-exposed facial skin, where chronic sun exposure markedly elevates IL-1ra while suppressing IL-1α [60,61]. In contrast, irritant-dose retinoic acid models show concurrent elevation of both IL-1ra and IL-1α as a compensatory pro-inflammatory response [62]. The observed decrease in IL-1α argues against irritant-driven activation as the dominant mechanism at 6 weeks. This pattern may instead reflect a temporal sequence: early retinol-induced keratinocyte activation transiently elevated both mediators, followed by progressive barrier normalization that suppressed IL-1α release while IL-1ra remained elevated. This interpretation is speculative given the single measurement timepoint. Since participants were instructed to apply broad-spectrum sunscreen throughout the study, UV-driven confounding cannot be fully excluded. Replication in strictly photoprotected cohorts with protein normalization is therefore warranted.

### 3.4. Dose-Response Relationship and Saturable Bioconversion

A key finding is the comparable efficacy of 0.104% and 0.247% formulations across most barrier function and biophysical parameters, with no statistically significant inter-group differences. This observation supports the concept of saturable retinol-to-retinoic acid bioconversion: enzymatic capacity (Lrat, Rdh12, Aldh1a3) and cellular binding protein availability (cellular retinoic acid-binding protein, CRABP; CRBP) limit the fraction of applied retinol that undergoes conversion to transcriptionally active RA [19]. Consequently, increasing topical concentration beyond a threshold does not proportionally enhance tissue-level outcomes, as only a finite amount permeates and reaches target receptors [19].

Our findings align with Zasada et al., who reported no significant differences between 0.3% and 0.5% retinol serums across multiple skin parameters [63,64]. Similarly, a pilot study comparing 0.15% and 0.3% retinol formulations demonstrated comparable improvements in skin hydration, color, and overall condition after 8 weeks, with no significant inter-group differences [65]. Jang et al. demonstrated that low-concentration retinol (1500–2500 IU) achieved superior outcomes for skin brightness and elasticity, while higher concentrations showed advantages for wrinkle reduction and dermal density after 24 weeks, suggesting parameter-specific dose-response relationships rather than a simple linear concentration–efficacy gradient [66]. Long-term studies (12–24 months) using stabilized 0.1% retinol have shown significant improvement in photoaging with favorable safety profiles, further supporting the clinical relevance of moderate dosing [34,67,68].

The absence of concentration-dependent differences has important implications for regenerative dermatology: clinically meaningful barrier restoration and structural remodeling can be achieved within restrictive EU safety limits (0.3% for facial products), minimizing exposure-related risks while maintaining therapeutic efficacy [37].

### 3.5. Tolerability and Safety Profile

Higher retinol concentration (0.247%) was associated with increased incidence of transient adverse effects: 35.7% reported skin condition worsening during initial treatment, and 28.6% experienced mild erythema and irritation. This finding is consistent with earlier studies demonstrating concentration-dependent irritation risk [5,16,38]. The lower concentration (0.104%) offered a favorable efficacy-to-tolerability profile, particularly relevant for sensitive skin or retinoid-inexperienced individuals [5,34].

The temporal dynamics of retinol action partially explain the absence of biomechanical parameter changes after 12 weeks. Proteomic studies indicate maximal epidermal retinol accumulation occurs after 3–6 months, while dermal structural changes become evident after approximately 12 months [14,20]. Initial therapy (0–3 months) primarily induces molecular changes (translation initiation factors EIF6, EIF2S1, EIF4A1), whereas visible structural effects—including improved elasticity—appear later [14,20]. Roman-Souza et al. observed elasticity improvements only after prolonged retinol use due to epidermal growth factor upregulation, suggesting our 12-week observation period was insufficient to detect biomechanical changes [53].

### 3.6. Translational Implications and Future Directions

This study provides evidence that topical retinol at analytically verified, EU-compliant concentrations induces tissue-level remodeling in mature human skin, supporting its role as a non-invasive regenerative approach [14,15,17]. The comparable efficacy of 0.104% and 0.247% formulations suggests that moderate concentrations may optimize the benefit-risk ratio, particularly for long-term use [34,64,67]. An exploratory observation of IL-1α reduction alongside IL-1ra elevation following 0.247% retinol treatment may reflect partial normalization of the age-related IL-1ra/IL-1α imbalance documented in mature skin [58,59]—with the IL-1ra increase potentially representing a counter-regulatory response to retinol-induced keratinocyte activation [69].

Future research should integrate serial cytokine monitoring with biophysical and proteomic assessments over extended periods (≥12 months). Beyond IL-1α and IL-1ra, a broader panel—including IL-18, tumor necrosis factor-alpha (TNF-α), and antimicrobial peptides—would allow more comprehensive characterization of retinol-induced epidermal immune modulation [20,51]. Additionally, advanced delivery systems such as solid lipid nanoparticles and nanostructured lipid carriers, combined with synergistic co-actives, may further optimize retinol’s stability and tolerability in clinical practice [12,30,70].

### 3.7. Study Limitations

Several limitations should be considered when interpreting these findings. The relatively short observation period (12 weeks for biophysical parameters, 8 weeks for ultrasound) may have precluded detection of longer-term dermal changes [14,20]. The main study lacked a placebo-controlled arm, as both groups received active retinol; this prevented assessment of spontaneous changes over time, though the split-face design in the ultrasound substudy partially addressed this. Sample size further limited several sub-analyses. The ultrasound substudy (*n* = 5) had limited statistical power, so structural results should be considered descriptive rather than inferential. The cytokine sub-study, conducted in a separate group of five participants, was similarly constrained by small sample size and additionally limited by the absence of total protein normalization, which may contribute to inter-individual variability in absolute IL-1α and IL-1ra values. Wrinkle assessment, though standardized, relies on semi-quantitative grading and may lack sensitivity to detect subtle improvements.

Several assessments also relied on indirect or retrospective methods rather than direct, prospective measurement. Retinol concentrations were verified by HPLC before the study, but retinol stability was not monitored during the 12-week treatment period. Opaque airless packaging and single-batch sourcing (Section 4.2) minimized oxidative exposure, but repeated HPLC quantification during treatment was not performed, so residual degradation cannot be excluded. Adverse effects were assessed retrospectively through a single end-of-study questionnaire referring to the initial treatment period, rather than a structured symptom diary or interim visits; precise onset and remission timepoints could therefore not be reconstructed. Similarly, the tight junction and cornified envelope mechanisms proposed to underlie the observed TEWL and barrier improvements are based on prior molecular literature [20,21], not on direct assessment in our cohort. This study did not include skin biopsies or protein-level analyses (e.g., immunohistochemistry or Western blotting); direct morphological or molecular confirmation of these changes remains an important direction for future studies.

## 4. Materials and Methods

### 4.1. Study Design and Participants

This prospective, randomized, single-blind study enrolled 38 women in total across three sub-studies. Twenty-eight participants (aged 40–61 years; mean age: 48.3 ± 6.2 years; Fitzpatrick phototypes II–III) were allocated to two groups (*n* = 14 per group) and assigned to use creams with different retinol concentrations—0.104% (HPLC-verified, labeled as 0.1%) or 0.247% (HPLC-verified, labeled as 0.3%)—for 12 weeks. Creams were applied to the face in pea-sized amounts (approximately 0.1–0.2 mL) each evening. Additionally, five women (aged 38–61 years; mean age: 44.8 ± 9.4 years) underwent a split-face ultrasound substudy, applying 0.247% retinol cream to the right cheek and retinol-free control cream to the left cheek for 8 weeks. A separate group of five participants (aged 43–59 years; mean age: 51.4 ± 5.7 years) underwent stratum corneum tape stripping for cytokine analysis following 6 weeks of 0.247% retinol treatment. All participants were instructed to apply broad-spectrum sunscreen (SPF ≥ 30) daily and to minimize direct sun exposure throughout the study period. The main study was conducted from November 2023 to May 2024; the cytokine sub-study was conducted in April–June 2026.

Inclusion criteria comprised visible signs of skin aging (wrinkles, loss of firmness and elasticity). Exclusion criteria included dermatological diseases, ongoing dermatologic treatments (topical or systemic), pharmacotherapy affecting study outcomes (e.g., corticosteroids, retinoids), pregnancy, lactation, and history of aesthetic procedures within defined timeframes: dermal fillers, radiofrequency, microfocused ultrasound, or laser resurfacing (within 1 year); botulinum toxin injections (within 8 months); chemical peels (within 2 weeks).

The study was conducted in accordance with the Declaration of Helsinki and approved by the Local Bioethics Committee of Poznan University of Medical Sciences (approval no. 977/22, 8 December 2022, for the main study and ultrasound substudy; and approval no. 282/26, 9 April 2026, for the cytokine sub-study). All participants provided written informed consent. Participants were blinded to the retinol concentration assigned (single-blind design).

### 4.2. Test Products

Three commercially available creams from the same EU manufacturer were used in this study: two retinol-containing formulations (analytically verified at 0.247% and 0.104% retinol by HPLC) with identical base composition, and one retinol-free control cream (used exclusively in the split-face ultrasound substudy). All creams were sourced simultaneously from a single production batch and supplied in opaque airless containers to minimize oxidative degradation. Complete INCI compositions are provided in Table 4 for transparency and reproducibility. The creams were intended for cosmetic use on facial skin and comply with EU Regulation (EC) No 1223/2009.

**Table 4:** INCI compositions of retinol and control creams used in this study.

Cream Type	Retinol Content	Commercial Origin	Full INCI Composition
Retinol Cream (0.3%)	0.247% retinol	Commercial, EU	Aqua, Coco-Caprylate/Caprate, Steareth-2, Glycerin, C30-H62, *Hippophae rhamnoides* (Seabuckthorn) Oil, Dimethicone, Hydroxypropyl Starch Phosphate, Cetearyl Alcohol, Steareth-21, Stearic Acid, Hydrolyzed Tomato Skin, Ascorbyl Glucoside, Sorbitol, *Boswellia serrata* Gum, Dihydroxy Methylchromone, Dipropylene Glycol, Trehalose, Urea, Sodium Citrate Dihydrate, Serine, Retinol, Pentylene Glycol, Glyceryl Polyacrylate, Algin, Carrageenan, Tetrasodium Glutamate Diacetate, Caprylyl Glycol, Sodium Hyaluronate, Pullulan, Polysorbate 20, Citric Acid, Benzyl Alcohol, Parfum, Dehydroacetic Acid, Disodium Phosphate, Potassium Phosphate, Potassium Hydroxide.
Retinol Cream (0.1%)	0.104% retinol	Commercial, EU	Same as above; identical INCI, only retinol concentration varies.
Control Cream (0%)	0% retinol	Commercial, EU	Aqua, *Vitis vinifera* Seed Oil, Propanediol, Saccharide Isomerate, Glycerin, Betaine, Glyceryl Stearate Citrate, *Butyrospermum parkii* (Shea Butter), *Adansonia digitata* Seed Oil, Glyceryl Stearate, Cetearyl Alcohol, Trolein, Parfum, Benzyl Alcohol, Glyceryl Polyacrylate, Trehalose, Urea, Sodium Hyaluronate, Carrageenan, Caprylyl Glycol, Algin, Citric Acid, Tetrasodium Glutamate Diacetate, Dehydroacetic Acid.

### 4.3. HPLC Analysis of Retinol Content

#### 4.3.1. Materials

Retinol (synthetic, ≥95% purity) was obtained from Sigma-Aldrich (St. Louis, MO, USA); catalog no. R7632. HPLC-grade methanol was obtained from Avantor Performance Materials Poland S.A. (Gliwice, Poland).

#### 4.3.2. HPLC Method and Validation

Retinol content was determined using a validated high-performance liquid chromatography (HPLC) method. Analyses were performed on a UHPLC Nexera-i LC-2040C system (Shimadzu, Kyoto, Japan) equipped with a Luna Omega C18 column (250 × 4.6 mm, 5 µm particle size, 100 Å pore size; Phenomenex, Aschaffenburg, Germany) and SecurityGuard™ C18 guard cartridges (4 × 3 mm; Phenomenex). Chromatographic separation was conducted under isocratic conditions using methanol:water (95:5, *v*/*v*) as mobile phase at a flow rate of 1.0 mL/min. The column temperature was maintained at 21.0 °C, UV detection was set at 326 nm, and injection volume was 10 µL.

The method was validated for linearity (r^2^ > 0.999), precision (intra-day and inter-day RSD < 2%), accuracy (recovery 98–102%), and sensitivity. Limits of detection (LOD) and quantification (LOQ) were 0.0357 µg/mL and 0.1082 µg/mL, respectively. Detailed validation parameters are provided in Table A1 (Appendix A).

#### 4.3.3. Sample Preparation and Analysis

Accurately weighed cream samples (0.1 g) were transferred to 25 mL volumetric flasks, dispersed in methanol, and diluted to volume. Samples were vortexed until complete dispersion, filtered through 0.22 µm nylon syringe filters (Millipore, Billerica, MA, USA), and analyzed by HPLC-UV. All measurements were performed in triplicate. HPLC analysis confirmed retinol content of 0.247 ± 0.007 mg/mL (labeled 0.3%) and 0.104 ± 0.001 mg/mL (labeled 0.1%).

### 4.4. Study Protocol and Conditions

The study was conducted under controlled laboratory conditions at 22–23 °C and 52–58% relative humidity. Measurements were performed at baseline and after 12 weeks (biophysical parameters) or 8 weeks (ultrasound substudy). Participants attended visits without makeup and refrained from washing or applying facial products for at least 3 h prior to assessment. A 30-min acclimatization period was mandatory before measurements.

### 4.5. Biophysical and Biomechanical Assessments

Skin parameters were evaluated using the Multi Probe Adapter MPA-9 system (Courage + Khazaka Electronic GmbH, Cologne, Germany) with the following probes: Corneometer^®^ CM 825: hydration (arbitrary units); Tewameter^®^ TM 300: transepidermal water loss (g/m^2^h); Mexameter^®^ MX 18: melanin and erythema indices; Skin-pH-Meter^®^ PH 905: skin surface pH; Colorimeter^®^ CL 400: skin brightness (L*, a, b values); Sebumeter^®^ SM 815: sebum level (µg/cm^2^) [71].

Measurements were performed on the central cheek area (2 cm below the infraorbital rim) in triplicate at adjacent points with participants in a supine position.

Biomechanical parameters (elasticity, viscoelasticity) were assessed using the CutiScan CS 100 (Courage + Khazaka Electronic GmbH) [71] on the lateral cheek, 1–2 cm below the oral commissure, using the suction method.

### 4.6. Wrinkle Assessment

Dynamic wrinkles were assessed using the Skin Aging Atlas photographic reference scales [47]. Standardized photography was performed under controlled lighting. Region-specific grading scales were applied: periocular wrinkles (crow’s feet, 0–6 scale), nasolabial folds (0–5 scale), glabellar and horizontal forehead wrinkles (0–5 scale each), and perioral wrinkles (0–4 scale). Upon study completion, participants completed a questionnaire assessing self-perceived changes in skin brightness, elasticity, smoothness, and hydration. Wrinkle grading was performed by a single trained dermatologist blinded to treatment allocation.

### 4.7. High-Frequency Ultrasound Imaging

In a substudy of five participants, 22 MHz high-frequency ultrasound imaging was performed using the Ultrascan UC 22 system (Courage + Khazaka Electronic GmbH, Cologne, Germany) [71]. The device generates B-mode scans in 256 grayscale levels, enabling non-invasive visualization of skin structures to a depth of 6–8 mm [52]. Measurements were obtained at the central cheek (2 cm below the infraorbital rim) at baseline and after 8 weeks. Epidermal thickness, epidermal density, dermal thickness, and dermal density were quantified using dedicated software provided with the system [51].

### 4.8. Stratum Corneum Tape Stripping and Cytokine Analysis

Stratum corneum sampling was performed on the left cheek of five participants, at a standardized site located 2 cm lateral to the nasal ala, marked with a cosmetic pencil to ensure identical sampling area across visits. On the morning of sampling, participants were instructed not to wash the face or apply any topical products. The skin was left untreated for at least 30 min before collection. Five consecutive Corneofix^®^ F20 adhesive tape strips (Courage + Khazaka Electronic GmbH, Cologne, Germany) were applied to the marked site and removed sequentially, each with uniform pressure applied for 5 s [42,72]. Tape 1 was discarded; Tape 2 was used for IL-1α analysis; Tapes 3–5 were used for IL-1ra analysis [43]. Each strip was immediately transferred into a separate 2.0 mL microcentrifuge tube.

Protein extraction was performed using 1.0 mL ice-cold phosphate-buffered saline (PBS, pH 7.2–7.4). Tape 2 was sonicated in PBS on ice for 15 min. For Tapes 3–5, a sequential extraction protocol was applied: each tape was successively transferred into the same supernatant and sonicated for 5 min under identical conditions. Following the final sonication step, samples were centrifuged (10,000× *g*, 5 min, 4 °C) and the clear supernatant was collected for analysis (100 µL per sample).

Cytokine concentrations were determined using commercial sandwich ELISA kits (Wuhan Fine Biotech Co., Ltd., Wuhan, China): Human IL-1α ELISA Kit (FineTest, Cat. No. EH0184) and Human IL-1ra/IL-1F3 ELISA Kit (FineTest, Cat. No. EH0172), performed according to the manufacturer’s instructions. Samples were measured in duplicate. Absorbance was read at 450 nm with background correction at 570 nm using an Epoch microplate spectrophotometer (BioTek, Winooski, VT, USA). Cytokine concentrations were calculated from standard curves using a four-parameter logistic (4-PL) regression model and expressed as pg/Tape (IL-1α) or pg per three sequential tapes (pg/3sT; IL-1ra).

### 4.9. Statistical Analysis

Data normality was assessed using the Shapiro–Wilk test. Baseline comparisons between groups were performed using Student’s *t*-test for independent samples. Within-group changes over time and group-by-time interactions were analyzed using two-way ANOVA. Between-group comparisons (0.104% vs. 0.247%) were conducted using independent *t*-tests after confirmation of variance homogeneity (Levene’s test). Analysis of variance (ANOVA) was used for inter-group comparisons across timepoints. Statistical significance was set at *p* < 0.05. For the tape stripping sub-study (*n* = 5), the Wilcoxon signed-rank test with exact two-sided *p*-values and Mid-*p* correction was applied. All analyses were performed using Statistica v.10.0 (StatSoft, Inc., Tulsa, OK, USA).

## 5. Conclusions

This prospective study demonstrates that topical retinol at analytically verified concentrations of 0.104% and 0.247% induces measurable barrier restoration and structural remodeling in mature facial skin. Both concentrations significantly improved transepidermal water loss, hydration, brightness, and sebum levels, and decreased skin surface pH, with ultrasound evidence of increased epidermal thickness and dermal density. Despite a more than two-fold difference in retinol concentration, efficacy was comparable between formulations. This supports saturable retinol-to-retinoic acid bioconversion, in which enzymatic and receptor-binding capacity, not applied dose, limits the biologically active signal within current EU safety limits.

Analytical pre-study verification of retinol content by HPLC addressed a critical regulatory gap, reinforcing the importance of independent quality control in cosmetic retinoid research. Lower-concentration retinol (0.104%) may be recommended as initial therapy for sensitive or retinoid-inexperienced individuals, offering a favorable efficacy-to-tolerability balance. Gradual titration to 0.247% may be considered in tolerant individuals.

An exploratory tape stripping sub-study provided preliminary evidence of stratum corneum cytokine modulation following sustained 0.247% retinol treatment, suggesting immunomodulatory activity concurrent with barrier improvement. To our knowledge, this represents the first such cytokine readout at EU-compliant cosmetic retinol concentrations.

These findings support topical retinol as a non-invasive, molecularly defined approach to cutaneous tissue regeneration. Future studies with extended follow-up (≥12 months), larger cohorts, integrated proteomic profiling, and cytokine monitoring are warranted to fully characterize retinol’s regenerative and immunomodulatory potential in mature skin.

## Figures and Tables

**Table 1:** Changes in biophysical skin parameters at baseline and after 12 weeks of treatment with 0.104% and 0.247% retinol creams.

Parameter (Mean ± SD)	0.104% Retinol		0.247% Retinol		*p* (Time)
	Baseline	Post-Therapy	Baseline	Post-Therapy	
Corneometer [AU]	56.88 ± 8.16	63.55 ± 6.47	57.67 ± 8.46	62.49 ± 7.51	**0.001**
Tewameter [g/m^2^/h]	14.54 ± 2.87	12.12 ± 3.04	14.96 ± 3.02	13.06 ± 3.20	**<0.001**
L* (skin brightness)	64.07 ± 3.29	67.80 ± 3.27	64.70 ± 1.88	66.78 ± 1.65	**<0.001**
a* (red-green) ^(a)^	10.31 ± 1.15	10.17 ± 1.14	10.45 ± 1.09	10.48 ± 1.22	0.768
b* (yellow-blue) ^(b)^	12.49 ± 1.49	12.10 ± 1.05	12.81 ± 1.35	12.10 ± 0.89	0.064
ITA [°] ^(c)^	51.21 ± 3.47	53.87 ± 3.72	49.50 ± 2.68	53.22 ± 3.95	**<0.001**
sebum [μg/cm^2^]	157.21 ± 46.66	118.71 ± 45.43	149.64 ± 43.82	109.14 ± 33.41	**<0.001**
pH	5.41 ± 0.35	5.22 ± 0.36	5.45 ± 0.41	5.24 ± 0.35	**0.002**

AU = arbitrary units; SD = standard deviation; ITA = Individual Typology Angle. *p*-values reflect the time effect from two-way ANOVA (cream × time); *p* < 0.05 was considered statistically significant. No significant between-group differences were detected. ^(a)^ a* (red-green axis): positive values indicate erythema/redness. ^(b)^ b* (yellow-blue axis): positive values indicate yellow/pigmentation tone. ^(c)^ ITA° calculated from L* and b*; higher values indicate lighter skin phototype. Bold values indicate statistically significant results (*p* < 0.05).

**Table 2:** Ultrasound-assessed epidermal and dermal parameters at baseline and after 8 weeks of split-face treatment with 0.247% retinol versus control cream (*n* = 5).

Parameter (Mean ± SD)	0.247% Retinol		Control Cream	
	Baseline	Post-Therapy	Baseline	Post-Therapy
epidermal thickness [μm]	137.6 ± 9.6	150.0 ± 18.3	140.6 ± 17.8	140.6 ± 11.3
epidermal density [%]	55.66 ± 4.51	59.50 ± 2.64	55.19 ± 4.98	54.56 ± 5.11
dermal thickness [μm]	1439.6 ± 224.5	1446.2 ± 201.6	1434.6 ± 238.5	1422.8 ± 224.3
dermal density [%]	12.53 ± 2.31	14.34 ± 2.37	12.00 ± 2.46	12.46 ± 2.58

All values are mean ± standard deviation (SD). No statistical tests were performed due to small sample size; results represent descriptive trends.

**Table 3:** Skin Aging Atlas wrinkle scores at baseline and after 12 weeks of treatment with 0.104% and 0.247% retinol creams (*n* = 14 per group).

Parameter (Mean ± SD) [Allergan Scale]	0.104% Retinol		0.247% Retinol	
	Baseline	Week 12	Baseline	Week 12
periocular wrinkles (crow’s feet, 0–6)	3.2 ± 0.8	2.7 ± 0.9	3.3 ± 0.7	2.6 ± 0.8
glabellar wrinkles (0–5)	2.4 ± 0.9	2.1 ± 0.8	2.5 ± 0.8	2.0 ± 0.8
horizontal forehead wrinkles (0–5)	2.6 ± 0.7	2.4 ± 0.7	2.7 ± 0.6	2.3 ± 0.7
perioral wrinkles, upper lip (0–4)	1.8 ± 0.6	1.7 ± 0.6	1.9 ± 0.7	1.7 ± 0.6
nasolabial folds (0–5)	3.1 ± 0.7	2.8 ± 0.8	3.2 ± 0.8	2.8 ± 0.7

All values are mean ± SD. Wrinkle severity was graded using the Skin Aging Atlas (Allergan scale) [47]. No statistically significant within-group or between-group changes were observed (all *p* > 0.05, Wilcoxon signed-rank test).

## Data Availability

The data presented in this study are available on request from the corresponding author. The data are not publicly available due to privacy restrictions related to the clinical study participants.

## Supplementary Materials

The following supporting information can be downloaded at: https://www.mdpi.com/article/10.3390/ijms27167205/s1.

## Abbreviations

The following abbreviations are used in this manuscript:

4-PL	Four-Parameter Logistic regression model
CRABP	Cellular Retinoic Acid-Binding Protein
CRBP	Cellular Retinol-Binding Protein
CTGF	Connective Tissue Growth Factor
EIF6	Eukaryotic Translation Initiation Factor 6
ELISA	Enzyme-Linked Immunosorbent Assay
HPLC	High-Performance Liquid Chromatography
IL-1α	Interleukin-1 Alpha
ITA°	Individual Typological Angle
IU	International Unit
LOD	Limit of Detection
LOQ	Limit of Quantification
Lrat	Lecithin Retinol Acyltransferase
MMPs	Matrix Metalloproteinases
NLC	Nanostructured Lipid Carriers
Nrf2	Nuclear Factor-E2-Related Factor 2
pg/Tape	picograms per tape
RAR	Retinoic Acid Receptor
Rdh12	Retinol Dehydrogenase 12
RXR	Retinoid X Receptor
SCCS	Scientific Committee on Consumer Safety
SD	Standard Deviation
SLN	Solid Lipid Nanoparticles
TEWL	Transepidermal Water Loss
TRP-1/TRP-2	Tyrosinase-Related Protein 1/2
Aldh1a3	Aldehyde Dehydrogenase 1 Family Member A3
ECM	Extracellular Matrix

## Appendix A

Concentration range for the method: 0.05–20 µg/mL.

**Table A1:** Summary of validation parameters for HPLC-UV methods of retinol determination in methanol.

Validation parameter	Medium–methanol		
Specifity	Yes		
Linearity *	Y = 59,774x (r = 0.9995)		
Limit of detection (LOD) ^(a)^	0.0357 μg/mL		
Limit of quantification ^(b)^	0.1082 μg/mL		
Accuracy (%) ^(c)^	20	μg/mL	4.18 ± 0.96
	10	μg/mL	5.68 ± 7.26
	5	μg/mL	7.19 ± 5.43
	2	μg/mL	7.90 ± 7.11
	1	μg/mL	8.60 ± 6.21
	0.75	μg/mL	8.45 ± 3.34
	0.50	μg/mL	0.95 ± 0.73
	0.25	μg/mL	3.10 ± 0.36
	0.10	μg/mL	9.26 ± 6.92
	0.05	μg/mL	13.51 ± 11.23
Precision (%) ^(d)^	20	μg/mL	0.22 ± 0.14
	10	μg/mL	0.06 ± 0.06
	5	μg/mL	0.12 ± 0.07
	2	μg/mL	0.34 ± 0.31
	1	μg/mL	0.23 ± 0.12
	0.75	μg/mL	0.76 ± 0.54
	0.50	μg/mL	0.95 ± 0.28
	0.25	μg/mL	1.25 ± 0.99
	0.10	μg/mL	2.68 ± 1.37
	0.05	μg/mL	4.52 ± 1.99

*—The calibration curve was constructed using linear regression with the intercept fixed at zero, as the intercept was not statistically significant (*p* > 0.05). ^(a)^ Calculated based on the standard deviation of the blank response, using the formula: LOD = 3.3 × (SD of blank/slope); ^(b)^ Calculated based on the standard deviation of the blank response, using the formula: LOD = 10 × (SD of blank/slope); ^(c)^ Expressed as the error of determination in % = |(mean determined concentration − nominal concentration)/(nominal concentration)| × 100%; ^(d)^ Expressed as coefficient of variation CV [%] = (standard deviation of determined concentrations)/(mean determined concentration) × 100%.
