# Supplementary material for: Barrier Function and Biophysical Effects of 0.104% and 0.247% Retinol Creams in Mature Facial Skin: A Prospective Study
Source: Int J Mol Sci. 2026 Aug 12;27(16):7205. doi: 10.3390/ijms27167205 (PMC13513334; doi:10.3390/ijms27167205)
Supplement: Supplementary file 1 [file ijms-27-07205-s001.zip › ijms-4479843-supplementary.pdf]

Supplementary Materials

Barrier Function and Biophysical Effects of 0.104% and 0.247% Retinol Creams in Mature Facial Skin: A Prospective Study

Iwona Pordąb [1,*], Julia Cieślawska [1], Michał Gackowski [2,3], Michał J. Kowalczyk [4], Małgorzata Pawłowska [5], Justyna Gornowicz-Porowska [1], Tomasz Osmałek [3], Marta Marzec [5], Izabela Nowak [5], Anna Kroma-Szal [1] and Mariola Pawlaczyk [1]

[1] Department and Division of Practical Cosmetology and Skin Diseases Prophylaxis, Faculty of Pharmacy, Poznan University of Medical Sciences, 3 Rokietnicka Street, 60-806 Poznań, Poland; justyna.gornowicz-porowska@ump.edu.pl (J.G.-P.); mariolapawlaczyk@ump.edu.pl (M.P.)
[2] Doctoral School, Poznań University of Medical Sciences, 70 Bukowska Street, 60-812 Poznań, Poland
[3] Department of Pharmaceutical Technology, Poznan University of Medical Sciences, 3 Rokietnicka Street, 60-806 Poznań, Poland
[4] Department of Dermatology and Venereology, Poznan University of Medical Sciences, 49 Przybyszewskiego Street, 60-355 Poznań, Poland; michalkowalczyk@ump.edu.pl
[5] Department of Applied Chemistry, Faculty of Chemistry, Adam Mickiewicz University, Poznań, 8 Uniwersytetu Poznańskiego Street, 61-614 Poznań, Poland; pawlowskamalgorzata@op.pl (M.P.); marta.dabrowska@amu.edu.pl (M.M.); nowakiza@amu.edu.pl (I.N.)
* Correspondence: pordab@ump.edu.pl

Table of Contents:

Table S1. Baseline comparison between the two retinol creams

Table S2. Two-way repeated-measures ANOVA (Cream × Time)

Table S3. Post-treatment comparison between creams

Table S1. Descriptive statistics and between-group comparison of all biophysical parameters at baseline (before treatment). All variables were normally distributed; groups were compared using the independent-samples t-test.

Parameter (baseline)	Cream	*N*	Mean	Median	Min	Max	SD	*p**
Corneometrer [AU]	0.247% retinol	14	57,67	55,54	47,22	72,00	8,46	0.8055
	0.104% retinol	14	56,88	56,30	40,00	70,83	8,16	
Tewameter [g/m²/h]	0.247% retinol	14	14,96	14,40	9,70	19,30	3,02	0.7086
	0.104% retinol	14	14,54	15,00	7,90	18,20	2,87	
L* (skin brightness)	0.247% retinol	14	64,70	64,94	60,45	67,58	1,88	0.539
	0.104% retinol	14	64,07	64,86	55,00	67,00	3,29	
a* (red-green) [(a)]	0.247% retinol	14	10,45	10,59	8,21	12,21	1,09	0.7499
	0.104% retinol	14	10,31	10,34	8,21	11,97	1,15	
b* (yellow-blue) [(b)]	0.247% retinol	14	12,81	12,72	10,29	15,08	1,35	0.5578
	0.104% retinol	14	12,49	12,54	9,42	14,57	1,49	
ITA [°] [(c)]	0.247% retinol	14	49,50	49,00	45,00	55,00	2,68	0.1552
	0.104% retinol	14	51,21	50,50	45,00	57,00	3,47	
sebum [μg/cm²]	0.247% retinol	14	149,64	149,50	83,00	209,00	43,82	0.6617
	0.104% retinol	14	157,21	167,50	83,00	220,00	46,66	
pH	0.247% retinol	14	5,45	5,45	4,98	6,40	0,41	0.7679
	0.104% retinol	14	5,41	5,40	4,78	5,98	0,35	

AU = arbitrary units; SD = standard deviation; ITA = Individual Typology Angle. *Independent-samples t-test (two-tailed), $\alpha = 0.05$. No statistically significant differences were observed between the 0.247% and 0.104% retinol groups at baseline for any parameter (all $p > 0.05$), confirming adequate group comparability prior to treatment. [(a)] a* (red-green axis): positive values indicate erythema/redness. [(b)] b* (yellow-blue axis): positive values indicate yellow/pigmentation tone. [(c)] ITA° calculated from L* and b*; higher values indicate lighter skin phototype.

Table S2. Results of the two-way ANOVA testing the main effects of cream concentration (0.247% vs. 0.104%), time (pre- vs. post-treatment), and their interaction, for each biophysical parameter. Since all parameters were normally distributed in both groups, ANOVA was used for the final comparative analysis.

Parameter	Cream (*p*)	Time (*p*)	Cream × Time (*p*)	Significant effect
Corneometrer [AU]	0.954	0.001	0.559	Time
Tewameter [g/m²/h]	0.544	<0.001	0.388	Time
L* (skin brightness)	0.810	<0.001	0.190	Time
a* (red-green) [(a)]	0.570	0.768	0.642	None
b* (yellow-blue) [(b)]	0.660	0.064	0.582	None
ITA [°] [(c)]	0.298	<0.001	0.459	Time
sebum [μg/cm²]	0.525	<0.001	0.913	Time
pH	0.782	0.002	0.897	Time

AU = arbitrary units; SD = standard deviation; ITA = Individual Typology Angle. Bold red p-values indicate statistically significant effects ($p < 0.05$). Only the main effect of time was significant across most parameters (corneometry, TEWL, L*, ITA, sebumetry, skin pH), indicating improvement over the course of treatment regardless of retinol concentration. No significant cream × time interaction was found for any parameter, indicating that the two concentrations produced comparable improvement over time. [(a)] a* (red-green axis): positive values indicate erythema/redness. [(b)] b* (yellow-blue axis): positive values indicate yellow/pigmentation tone. [(c)] ITA° calculated from L* and b*; higher values indicate lighter skin phototype.

Table S3. Independent-samples *t*-test comparing post-treatment values between the 0.247% and 0.104% retinol creams for each biophysical parameter.

Parameter (post-treatment)	Test used	*p* (between creams)
Corneometrer [AU]	Independent-samples *t*-test	0.6912
Tewameter [g/m²/h]	Independent-samples *t*-test	0.4316
L* (skin brightness)	Independent-samples *t*-test	0.3100
a* (red-green) [(a)]	Independent-samples *t*-test	0.4878
b* (yellow-blue) [(b)]	Independent-samples *t*-test	0.9923
ITA [°] [(c)]	Independent-samples *t*-test	0.6612

sebum [μg/cm²]	Independent-samples *t*-test	0.5309
pH	Independent-samples *t*-test	0.8364

AU = arbitrary units; SD = standard deviation; ITA = Individual Typology Angle. No statistically significant differences were found between the two retinol concentrations for any parameter at the post-treatment time point (all $p > 0.05$), indicating that both concentrations produced a comparable overall biophysical outcome by the end of the study. [a] a* (red-green axis): positive values indicate erythema/redness. [b] b* (yellow-blue axis): positive values indicate yellow/pigmentation tone. [c] ITA° calculated from L* and b*; higher values indicate lighter skin phototype.
